# On Optimal Cooperative Sensing with Energy Detection in Cognitive Radio

**DOI:** 10.3390/s17092111

**Published:** 2017-09-15

**Authors:** Sunghwan Bae, Jaewoo So, Hongseok Kim

**Affiliations:** Department of Electronic Engineering, Sogang University, Seoul 04107, Korea; usher731@sogang.ac.kr (S.B.); jwso@sogang.ac.kr (J.S.)

**Keywords:** cognitive radio, cooperative sensing, energy detection, globally optimal threshold, hard decision

## Abstract

In this paper, we propose an optimal cooperative sensing technique for cognitive radio to maximize sensing performance based on energy detection. In most spectrum sensing research, many cooperation methods have been proposed to overcome the sensitivity of energy detection so that both primary and secondary users are better off in terms of spectral efficiency. However, without assigning a proper sensing threshold to each sensing node, cooperation may not be effective unless the received average primary user signal-to-noise ratio (SNR) is *identical*. We show that equal threshold energy detection severely degrades sensing performance when cooperative sensing nodes experience diverse average SNRs, and it is not unusual for even single-node sensing to be better than cooperative sensing. To this end, based on the Neyman–Pearson criterion we formulate an optimization problem to maximize sensing performance by using optimized thresholds. Since this is a non-convex optimization problem, we provide a condition that convexifies the problem and thus serves as a sufficient optimality condition. We find that, perhaps surprisingly, in all *practical* cases one may consider this condition satisfied, and thus optimal sensing performance can be obtained. Through extensive simulations, we demonstrate that the proposed technique achieves a globally optimal solution, i.e., it maximizes the probability of detection under practical operating parameters such as the target probability of false alarm, different SNRs, and the number of cooperative sensing nodes.

## 1. Introduction

We are entering the era of a fourth industrial revolution where both hyper-connectivity and digital automation enable a large number of different types of economic entities to share information quickly for optimal decision-making based on industrial IoT sensor networks. This wave has driven 5G New Radio (NR) development to provide greater capacity and higher reliability for tactile internet, artificial intelligence (AI), and smart factory applications, etc. Since intelligent and efficient ways of utilizing scarce spectrum are required to meet the huge demand for increasing capacity, new, emerging technologies for dynamic spectrum management have been developed to improve spectral efficiency.

Cognitive radio (CR) is a promising form of technology for combating spectrum scarcity in next-generation wireless networks. A group of secondary users (SUs) can exploit the unused spectrum bands of a primary user (PU) in an opportunistic manner as long as no harmful interference is guaranteed [1]. Recent worldwide migration from analog to digital television broadcasting has expedited sharing of underutilized spectrum bands called TV white space (TVWS) [2]. For example, standards such as IEEE 802.22 and IEEE 802.11af opportunistically utilize TVWS, and regulatory bodies such as the FCC and OFCOM have updated rules for TVWS and other unlicensed bands [3]. TVWS-based CR was also introduced to enable smart grid communications in rural areas because of the good propagation properties of TVWS [4]. LTE-CR was proposed as a cellular application to extend the LTE time-division duplex (TDD) to support TVWS [5]. In addition to TVWS, 3GPP has developed LTE in an unlicensed spectrum so that LTE can harmoniously coexist with other wireless systems in a 5-GHz spectrum [6].

To support CR, one of the key roles of SUs is to sense the PU with high detection probability and evacuate the PU’s band immediately. There are several detection techniques, such as energy detection, matched filter detection, cyclostationary detection, and compressive sensing, [7]. Among these techniques, energy detection of [8] has been widely adopted in practice because it has low complexity and does not require prior knowledge of the PU [9]. However, since sensing performance of energy detection can deteriorate when the signal-to-noise ratio (SNR) is low [10], many cooperative sensing techniques have been proposed to overcome performance degradation [11,12,13,14,15,16,17,18,19,20,21,22,23,24].

Specifically, to maximize sensing performance, a series of cooperation algorithms have been investigated in terms of sensing duration, the number of cooperative sensing nodes, and thresholds of energy detection [11,12,13,14,15,16,17,18]. In [11], sensing-throughput tradeoff depending on sensing duration was investigated. The author of [12] jointly optimized sensing time and reporting time to maximize throughput based on maximal ratio combination (MRC)-based soft decision. The fundamental performance limits of the cooperative sensing using energy detection were analyzed by considering the unlimited number of sensing nodes, and it has been shown that the OR rule achieves *zero* false alarm probability for any given target detection probability irrespective of the received PU SNR [13]. In [14], the authors proposed optimal counting rules of hard decision under the Neyman–Pearson criterion and Bayesian criterion, respectively. In [15], both sensing duration and fusion rule were jointly optimized to maximize the throughput of SUs under the constraint of the probability of detection. In [16,17], an optimal *k* was investigated for the so called *k*-out-of-*N* rule, and then the *same* threshold for all sensing nodes is optimized by a given *k*. The authors of [18] proposed an on/off reporting mechanism to achieve robust cooperative sensing over an imperfect reporting channel as well as graceful degradation against sensor failures.

However, the existing work [11,12,13,14,15,16,17,18] assumed that the average SNR is *identical* for all cooperative sensing nodes, and so is the sensing threshold. This assumption may not be realistic in practical systems. In addition to multipath fading and shadowing effects, there are several practical factors for heterogeneous cooperative sensing, such as geographically different positions (indoor or outdoor), inherent RF front-end sensitivity, obstacles, temperature variations, and so on. Thus, cooperative sensing performance highly depends on the heterogeneity of the received PU SNRs at individual SUs. Although previous work, such as [19], considered heterogeneous average SNRs for cooperative sensing nodes, all sensing nodes still used the same sensing threshold.

In addition, the authors in [20,21,22,23,24] considered optimizing thresholds of cooperative sensing nodes based on different average SNRs. In [20], an iterative threshold selection algorithm was proposed to determine whether a specific sensing node can participate in cooperation or not, based on its contribution to detection performance. The work of [21] proposed a weighted decision rule by jointly optimizing the test threshold of likelihood-ratio and the local threshold of SUs. Although heuristic methods of optimizing two thresholds at a time did not guarantee the achievement of global optimal solutions, the proposed algorithm provided the highest throughput results over the non-jointly optimized schemes [21]. In [22,23], a heuristic algorithm was proposed to achieve suboptimal thresholds at cooperative sensing nodes by minimizing false alarm probability subject to target missed detection probability. Different fusion rules with iterative algorithms were proposed to decrease the total error detection probability for both single channel and multichannel cooperative sensing [24].

However, even though the existing literature [20,21,22,23,24] has investigated distinct thresholds for cooperative sensing nodes according to their own average SNRs, they are suboptimal and *theoretically* achieving the optimal detection performance still remains unsolved. Moreover, previous heuristic approaches would suffer from high computational complexity as the number of cooperative sensing nodes increases [20,21,22,23,24]. In this regard, we are motivated to theoretically develop a globally optimal solution for energy detection under a heterogeneous PU SNR environment.

We summarize our key contributions as follows. Based on the Neyman–Pearson criterion we formulate an optimization problem to maximize the sensing performance when a hard decision is used under different average SNRs. It is found that this is a non-convex optimization problem, and thus we provide a condition that convexifies the original problem. We then verify that all *practically meaningful* parameters satisfy the convexifying condition. Thus, our solution guarantees the maximized detection performance of cooperative sensing. Considering that using a single common threshold severely degrades the sensing performance when cooperative sensing nodes experience different average SNRs, our solution is promising. Our extensive simulations show that a globally optimized solution achieves an improvement of several orders of magnitude improvement under practical operating parameters such as the target probability of false alarm, different average SNRs, and the number of cooperative sensing nodes.

The rest of this paper is organized as follows. In Section 2, the CR system model and problem formulation are introduced. Cooperative sensing with optimal thresholds is presented with a sufficient optimality condition in Section 3. We present numerical results under practical operating parameters in Section 4. Finally, we draw our conclusions in Section 5.

## 2. System Model

In this section, we provide a scenario of cooperative sensing operation in the CR system. Using energy detection and its sensing performance metrics, we formulate an optimization problem.

### 2.1. Cognitive Radio System

**Figure 1:**
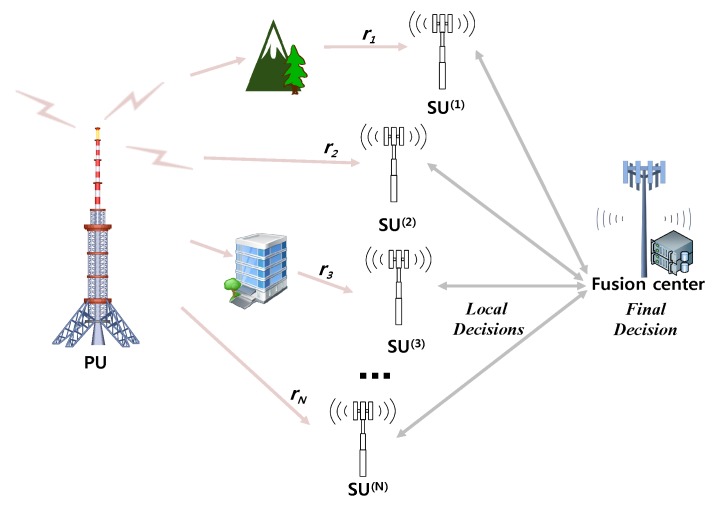
A scenario for cooperative sensing of cognitive radio system in wireless networks; there is a primary user, cooperative secondary users, and a fusion center. SU: secondary user.

Figure 1 illustrates a scenario for CR system with cooperative sensing in wireless networks. A SU can temporally and spatially utilize the licensed or unlicensed spectrum band of the PU. To avoid possible interference to the PU, the CR system requires SUs to simultaneously perform in-band sensing, which means SUs periodically sense the operating spectrum band to verify whether the PU appears or not. In addition to in-band sensing, out-of-band sensing implies that SUs sense the non-operating bands and maintain a list of available bands. Even though our work is applicable to both in-band and out-of-band sensing purposes, hereafter we consider in-band sensing. When SUs finish sensing, they send their own sensing results to a fusion center so that it combines all local results and makes a final decision about the presence of PUs. We assume that SUs honestly report their local decisions to the fusion center via common control channels [11,12,13,14,15,16,17], e.g., an individual reporting channel is reliable and error-free for simplicity [19,20,21,22,23,24].

### 2.2. Energy Detection and Sensing Performance Metrics

Let τ be a continuous value denoting sensing duration. Under the absence of PU (hypothesis $H_{0}$), the received signal at a sensing node is given by

r(t)=n(t),

where n(t) is the noise power at time *t*. Under the presence of PU (hypothesis $H_{1}$), we have

r(t)=h(t)·s(t)+n(t),

where s(t) is the received PU signal and h(t) is the channel gain at time *t*. We assume that n(t) is white Gaussian noise with two-sided power spectral density $N_{0}$*(AS I)*, and sensing duration τ is short so that the channel can be assumed to be static *(AS II)*. According to [8], the test statistic using energy detection is given by

$$T=\frac{1}{N_{0}}\int_{0}^{\tau }{\left|r\left(t\right)\right|}^{2}dt.$$

Let γ be the received SNR given by $\gamma =\frac{P}{N_{0}B}$, where *P* denotes the received power of PU at a sensing node, and *B* is the channel bandwidth. Under the assumptions of *AS I* and *AS II*, the received energy of PU signal is simply Pτ. When PU is absent, *T* has the central chi-square distribution with the degrees of freedom equal to 2τB. When PU is present, *T* has the non-central chi-square distribution with 2τB degrees of freedom and non-centrality parameter $\delta =\frac{P\tau }{N_{0}}$ equal to γτB. Based on the central limit theorem (CLT), when 2τB is more than 250, *T* under two conditions can be considered as Gaussian random variables [8].

(1) $$T∼\left\{\begin{array}{cc} \;N\left(2\tau B,4\tau B\right), & \mathrm{under}\;H_{0},\\ \;N\left(2\tau B+\gamma \tau B,4\tau B+4\gamma \tau B\right), & \mathrm{under}\;H_{1}, \end{array}\right.$$

where $N\left(m,\sigma^{2}\right)$ represents the normal distribution with the mean *m* and the variance $\sigma^{2}$. From now on, we assume that 2τB is large enough so that the CLT condition is met, and thus *T* is a Gaussian random variable.

Suppose that we have *N* cooperative sensing nodes using energy detection. Let $\epsilon_{i}$ be the detection threshold and $P_{i}^{d}$ be the probability of detection, i.e., $P_{i}^{d}=P\left(Y\geq \epsilon_{i}|H_{1}\right)$ at a sensing node $i\in \left\{1,\cdots ,N\right\}$ [11,13]. Then, we have

(2) $$P_{i}^{d}\left(\epsilon_{i},\gamma_{i},\tau ,B\right)=Q\left(\frac{\epsilon_{i}-\left(2\tau B+\gamma_{i}\tau B\right)}{\sqrt{4\tau B+4\gamma_{i}\tau B}}\right),$$

where

(3) $$Q\left(t\right)=\frac{1}{\sqrt{2\pi }}\int_{t}^{\infty }\exp\left(\frac{-u^{2}}{2}\right)du.$$

Let $P_{i}^{f}$ denote the probability of false alarm, i.e., $P_{i}^{f}=P\left(Y\geq \epsilon_{i}|H_{0}\right)$ at a sensing node $i\in \left\{1,\cdots ,N\right\}$ [11,13], and we have

(4) $$P_{i}^{f}\left(\epsilon_{i},\tau ,B\right)=Q\left(\frac{\epsilon_{i}-2\tau B}{\sqrt{4\tau B}}\right).$$

We notice that there is no PU signal present under $H_{0}$, and then $P_{i}^{f}$ is independent of $\gamma_{i}$. However, when h(t) is varying due to shadowing/fading, $P_{i}^{d}$ gives the detection probability conditioned on the instantaneous SNR $\gamma_{i}$. In this case, the average detection probability can be derived by averaging (2) over fading statistics.

(5) $${\tilde{P}}_{i}^{d}=\int_{}^{}Q\left(\frac{\epsilon_{i}-\left(2\tau B+\gamma_{i}\tau B\right)}{\sqrt{4\tau B+4\gamma_{i}\tau B}}\right)f_{\gamma }\left(\gamma_{i}\right)d\gamma_{i},$$

where $f_{\gamma }\left(\gamma_{i}\right)$ is the probability distribution function (PDF) of SNR under fading/shadowing and its mean value is ${\bar{\gamma }}_{i}$ [25]. In addition to the aforementioned probabilities, the probability of miss detection is $P_{i}^{m}=1-\;P_{i}^{d}$, and the probability of correct rejection is $P_{i}^{c}=1-P_{i}^{f}$ at a sensing node $i\in \left\{1,\cdots ,N\right\}$. During cooperative sensing, a PU is assumed to be either present or absent. Although multiple PUs can arrive or depart randomly, this assumption is still reasonable based on the long time average scenario of observed PU behavior. One may refer to [26,27] to optimize both the sensing time and sensing period (duty cycle) so that instantaneous sensing performance improves under random arrivals of multiple PUs in cooperative sensing.

### 2.3. Problem Formulation of Cooperative Sensing

We investigate optimal sensing thresholds for sensing nodes using hard decision. Even though use of the soft decision achieves better performance than the hard decision, the burden of reporting overhead hinders the use of soft decision [28]. One may want to directly consider the *k*-out-of-*N* rule, but finding an globally optimal solution for the *k*-out-of-*N* rule is not very tractable unless the same average SNR value is assumed for cooperative sensing scenarios [16]. Moreover, [21,22,23] pointed out the very high computational complexity and impracticality of finding suboptimal *k*. Thus, we first focus on AND and OR rules in this paper. The fusion center using the AND rule decides that PU is present when all cooperative sensing nodes report PU presence to the fusion center [9]. Then, the final detection and the final false alarm probabilities at the fusion center are simply given by

$$P^{d}=\prod_{i=1}^{N}P_{i}^{d},$$

$$P^{f}=\prod_{i=1}^{N}P_{i}^{f}.$$

Based on the Neyman–Pearson criterion, our aim is to maximize the final detection probability $P^{d}$ subject to the target final false alarm probability $\bar{P^{f}}$, which is formulated as follows:

(6) $$\begin{array}{cc} \max_{\epsilon_{i},i\in \left\{1,\cdots ,N\right\}} & P^{d}=\prod_{i=1}^{N}P_{i}^{d},\\ s.t. & P^{f}=\prod_{i=1}^{N}P_{i}^{f}\leq \bar{P^{f}}.\; \end{array}$$

The fusion center using the OR rule decides that the PU is present when any cooperative sensing nodes report PU presence to the fusion center [9]. Then, the final detection and the final false alarm probabilities at the fusion center are simply

$$P^{d}=1-\prod_{i=1}^{N}\left(1-P_{i}^{d}\right),$$

$$P^{f}=1-\prod_{i=1}^{N}\left(1-P_{i}^{f}\right).$$

Then, we can also use the OR rule to maximize the final detection probability $P^{d}$ subject to the target final false alarm probability $\bar{P^{f}}$ as follows:

$$\begin{array}{cc} \max_{\epsilon_{i},i\in \left\{1,\cdots ,N\right\}} & P^{d}=1-\prod_{i=1}^{N}\left(1-P_{i}^{d}\right),\\ s.t. & P^{f}=1-\prod_{i=1}^{N}\left(1-P_{i}^{f}\right)\leq \bar{P^{f}}.\; \end{array}$$

Considering the symmetric mathematical structure of $P_{i}^{m}$ and $P_{i}^{c}$, an alternative optimization problem is formulated as follows:

$$\begin{array}{cc} \min_{\epsilon_{i},i\in \left\{1,\cdots ,N\right\}} & P^{m}=\prod_{i=1}^{N}P_{i}^{m},\\ s.t. & P^{c}=\prod_{i=1}^{N}P_{i}^{c}\geq \bar{P^{c}},\; \end{array}$$

where $\bar{P^{c}}$ is according given by $1-\bar{P^{f}}$.

If the globally optimal solution can be derived from the AND rule of (6), our optimization approach is also applicable to the OR rule because of its symmetric mathematical structure. AND and OR rules are two opposite extremes. For comparison, the existing literature [7,11,13,28] have shown that dominant performance regions of both rules highly depend on system parameters such as $\tau ,\bar{\gamma },B,$ and $\bar{P^{f}}$ for different purposes. Herein we mainly focus on the existence of globally optimized sensing thresholds. Therefore, in the rest of paper, our problem formulation of cooperative sensing mainly exploits the mathematical structure of the AND rule so that we can provide a globally optimal solution to maximize the detection performance of cooperative sensing under different average SNRs.

## 3. Cooperative Sensing with Optimal Thresholds

### 3.1. Limitation of Equal Threshold—A Motivating Example with Two Sensing Nodes

To better understand the effect of sensing thresholds, in this section we first verify the detection performance with both equal and optimal thresholds when only two cooperative sensing nodes experience different average SNRs.

**Figure 2:**
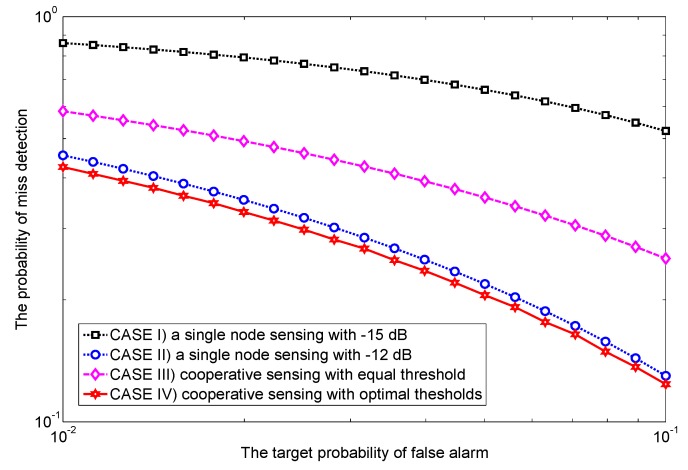
Receiver operating characteristic (ROC) curves of cooperative sensing with two sensing nodes; node 1 with −15 dB average signal-to-noise ratio (SNR) and node 2 with −12 dB SNR.

**Example** **1.** *Since the same average SNR for all sensing nodes in [11,13,14,15,16,17] may not be realistic in practice because of spatial diversity, herein we use different average SNRs in cooperative sensing [19,20,21,22,23,24]. As a motivating example, Figure 2 shows the receiver operating characteristic (ROC) curves under four different scenarios: the sensing performance of a single node sensing with the worst and the best average SNRs (CASE I and CASE II) as well as that of both equal and optimal thresholds under different average SNRs (CASE III and CASE IV). To obtain the equal threshold ϵ for two cooperative sensing nodes [19], the $P_{i}^{f}$ at individual sensing node is simply given by $\sqrt{\bar{P^{f}}}$ from *(6)*, and then ϵ is accordingly given by $\sqrt{4\tau B}Q^{-1}\left(\sqrt{\bar{P^{f}}}\right)+2\tau B$ from *(4)*. Interestingly, compared to a single node sensing with a higher SNR (−12 dB), the sensing performance of cooperative sensing with equal threshold is even worse, and thus cooperation is not beneficial at all. To overcome the degradation of the detection performance, we assign the optimal threshold to each sensing node based on an exhaustive search for illustrative purposes. By doing so, optimal thresholds can improve the sensing performance. Since an exhaustive search is not suitable for a large number of sensing nodes, in Section 3.2, we further develop a convex optimization algorithm to achieve optimal thresholds. When many sensing nodes cooperate, we are also interested in knowing how much cooperative gain is expected by using globally optimized sensing thresholds.*

**Figure 3:**
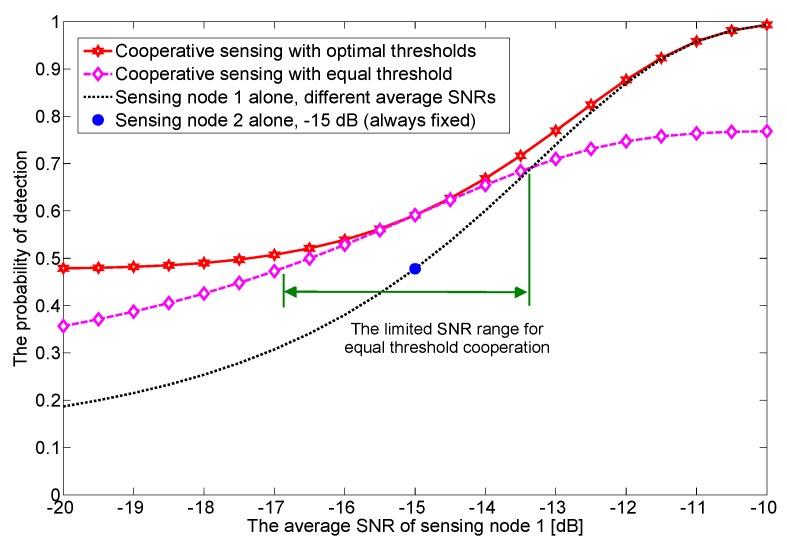
The detection performance for a single and two cooperative sensing cases when the average SNR of the sensing node 1 varies from −20 dB to −10 dB and that of the sensing node 2 is fixed at −15 dB. We notice that the effective range of the average SNR is very limited for the equal threshold case.

**Example** **2.** In Figure 3, we verify how the difference of average SNRs affects the detection performance of cooperative sensing. In this example with two sensing nodes, we vary the average SNR of one sensing node and fix that of the other sensing node. For the equal threshold case, we notice that the effective range of the average SNR is very limited. However, using optimal thresholds alway outperforms other cases regardless of the average SNR difference. To this end, along with the previous work [19,20,21,22,23,24], we are motivated to find out the globally optimal solution rather than suboptimal solutions. We will see that cooperative sensing dramatically (by several orders of magnitudes) improves sensing performance under practical operating parameters such as the target probability of false alarm, different average SNRs, and the number of cooperative sensing nodes.

### 3.2. Cooperative Sensing with Optimal Thresholds

In solving (6), the individual sensing threshold for sensing node *i* is $\epsilon_{i}=\sqrt{4\tau B}Q^{-1}\left(P_{i}^{f}\right)+2\tau B$ by (4). Then, we can use $P_{i}^{f}$ as optimization variables instead of $\epsilon_{i}$, and rewrite the optimization problem (6) by using (2) for analytical tractability:

(7) $$\begin{array}{cc} \max_{P_{i}^{f},i\in \left\{1,\cdots ,N\right\}} & \prod_{i=1}^{N}Q{\left(\frac{1}{\sqrt{1+\gamma_{i}}}\left(Q^{-1}\left(P_{i}^{f}\right)-\;\frac{\gamma_{i}\sqrt{\tau B}}{2}\right)\right)}_{,}\\ s.t. & \prod_{i=1}^{N}P_{i}^{f}\leq \bar{P^{f}}. \end{array}$$

With one further step, we define the *normalized* threshold $x_{i}≜\frac{\epsilon_{i}-2\tau B}{\sqrt{4\tau B}}$ from $H_{0}$ of (1). Then, it can be easily drawn from (4) such that $x_{i}=Q^{-1}\left(P_{i}^{f}\right)$.

After taking the logarithm of (7), the problem is equivalent to the following optimization problem:

(8) $$\begin{array}{cc} \max_{x_{i},\;y_{i},\;i\in \left\{1,\cdots ,N\right\}} & \sum_{i=1}^{N}\log Q\left(y_{i}\right),\\ s.t. & y_{i}=\frac{1}{\sqrt{1+\gamma_{i}}}\left(x_{i}-\;\frac{\gamma_{i}\sqrt{\tau B}}{2}\right),\;\\ & \sum_{i=1}^{N}\log Q\left(x_{i}\right)\leq \log\bar{P^{f}},\; \end{array}$$

where $y_{i}$ is introduced as an auxiliary variable. The convex problem has three requirements: the objective function must be concave for the maximization problem; the equality constraint functions must be affine; and the inequality constraint convex functions must be non-positive [29]. Note that although the objective function of (8) is concave, (8) still remains a non-convex optimization problem. This is because when we make the inequality constraint functions convex, it becomes non-negative which cannot satisfy a standard form of convex optimization. Thus, the problem cannot be posed as convex optimization [29]. Hence, we now try to find the condition that can convexify the problem as presented in the following proposition.

**Proposition** **1.** *Suppose that observations of cooperative sensing nodes follow the normal distribution as in *(1)* with different SNR $\gamma_{i}$. Then, based on the Neyman–Pearson criterion, *(8)* can be convexified if*

(9) $$R\left(x_{i}\right)+x_{i}\geq \frac{1}{\sqrt{1+\gamma_{i}}}\left(R\left(y_{i}\right)+y_{i}\right),\;\forall \;i\in \left\{1,\cdots ,N\right\},$$

*where a function R(t) is defined as $R\left(t\right)≜\frac{Q^{\prime }\left(t\right)}{Q(t)}$.*

**Proof.**  See Appendix A. ☐

**Remark** **1.** Note that Proposition 1 serves as a sufficient optimality condition. As long as the sufficient optimality condition is satisfied, the problem can be convexified so that a globally optimal solution can be obtained.

We notice that the sufficient optimality condition may not always hold for $\forall x_{i}\in R$. However, by considering all the practically meaningful scenarios, we rigorously verify that as long as $P_{i}^{f}$ is on [ε,1−ε] where ε is infinitesimally small and sensing observations follow the normal distribution, the sufficient optimality condition holds so that a globally optimal solution is guaranteed in cooperative sensing, as described in Examples 3 and 4.

**Figure 4:**
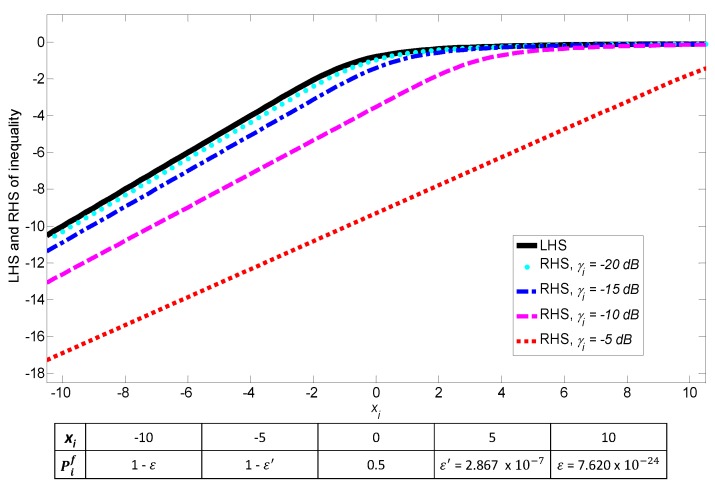
Under practical operating parameters, the y-axis is used to depict value for both left- and right-hand sides of inequality (9) when $x_{i}=Q^{-1}\left(P_{i}^{f}\right)$ varies. Correspondingly, the table is used to represent value of $P_{i}^{f}$. LHS: left-hand side; RHS: right-hand side.

**Figure 5:**
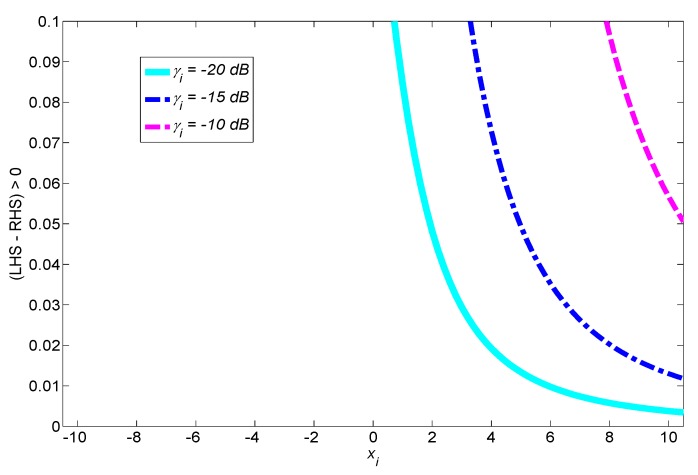
Inequality condition (9) in Proposition 1 holds for practical operating parameters.

**Example** **3.** *To investigate the practicality of Remark 1, in Figure 4 we plot both the left-hand side (LHS) and the right-hand side (RHS) of *(9)* with τ=1 ms and B=6 MHz for various PU SNR $\gamma_{i}$. As can be seen, LHS is greater than RHS, and thus *(9)* holds for $\forall x_{i}\in \left[-10,10\right]$. Recall that $x_{i}$ is a normalized version of $\epsilon_{i}$ that determines the false alarm probability, i.e., $P_{i}^{f}=Q\left(x_{i}\right)$ and $x_{i}$ has a region of interest depending on $P_{i}^{f}$. Ideally, $P_{i}^{f}$ should be any value on [0,1]. We notice that when *(9)* holds, the corresponding $P_{i}^{f}$ covers almost all values on (0,1). In this example, $P_{i}^{f}$ is in the interval [ε,1−ε] where $\varepsilon ≃7.620\times 10^{-24}$, and thus it is obvious that a set of $x_{i}$ satisfying *(9)* is a super set of the practical region of interest. In Figure 5, we also show that (LHS-RHS) is positive in *(9)*. Note that although the gap between LHS and RHS depends on the range of SNR, the inequality condition itself still holds as long as $P_{i}^{f}$ is on [ε,1−ε] regardless of SNR. For example, even for the worst case of zero SNR, the condition *(9)* holds because LHS becomes equal to RHS.*

**Example** **4.** *One may want to verify practicality of Remark 1 with other values of τ and B. Thus we also consider the worst case condition such that 2τB is small and just satisfies the minimum CLT condition of *(1)*, e.g., 2τB≃ 250. We confirm that when $x_{i}\geq -5$, *(9)* also holds and the corresponding $P_{i}^{f}$ spans in the interval $\left(0,1-\varepsilon^{\prime }\right]$ where $\varepsilon^{\prime }≃2.867\times 10^{-7}$. Thus, although the problem is a non-convex optimization, it can be convexified with Proposition 1 in practice, and a globally optimal solution can be derived as in the following subsection.*

### 3.3. Convex Optimization

Under the region of satisfying (9), now we can obtain the globally optimal solution by applying the standard convex optimization procedure. Let $v=(v_{1},\cdots ,v_{N})$, and the Lagrangian function of (A1) is

$$\begin{array}{ccc} L(v,\lambda ) & = & \sum_{i=1}^{N}\log Q\left(a_{i}\left({\left(\log Q\right)}^{-1}\left(v_{i}\right)-{\tilde{\gamma }}_{i}\right)\right)+\lambda \left(\log\bar{P^{f}}-\sum_{i=1}^{N}v_{i}\right), \end{array}$$

where λ is the Lagrange multiplier. The KKT condition for this problem consists of four parts, which are a primal feasible, dual feasible, complementary slackness, and zero gradient:

(10) $$\begin{array}{ccc} \sum_{i=1}^{N}v_{i}^{*}-\log\bar{P^{f}} & \leq & 0,\\ \lambda^{*} & \geq & 0,\\ \lambda^{*}\left(\sum_{i=1}^{N}v_{i}^{*}-\log\bar{P^{f}}\right) & = & 0,\\ \frac{Q^{\prime }\left(a_{i}\left({\left(\log Q\right)}^{-1}\left(v_{i}^{*}\right)-{\tilde{\gamma }}_{i}\right)\right)Q\left({\left(\log Q\right)}^{-1}\left(v_{i}^{*}\right)\right)a_{i}}{Q\left(a_{i}\left({\left(\log Q\right)}^{-1}\left(v_{i}^{*}\right)-{\tilde{\gamma }}_{i}\right)\right)Q^{\prime }\left({\left(\log Q\right)}^{-1}\left(v_{i}^{*}\right)\right)}-\lambda^{*} & = & 0,\;i=1,\cdots ,N. \end{array}$$

Since there is one Lagrange multiplier λ and also $v_{i}$ can be expressed by a function of λ according to (10), one can obtain the optimal $\left(v^{*},\lambda^{*}\right)$ by adjusting λ based on some iterative methods such as bisection method and Newton’s method. The details of the convex optimization algorithm are shown in Algorithm 1 based on the bisection method.

**Algorithm 1:** Find the globally optimal $v^{*}$ and $\lambda^{*}$ for (10).**Input:** Given *N*, τ, *B*, $\bar{P^{f}}$, and $\gamma_{i}$ for all *i*.  1:  Set initial $\lambda_{\min}$ and $\lambda_{\max}$.  2:  Calculate $a_{i}$ and ${\tilde{\gamma }}_{i}$ for all *i*.  3:  **while**
$|\lambda_{\min}-\lambda_{\max}|>\varepsilon$
**do**  4: $\lambda =(\lambda_{\min}+\lambda_{\max})/2.$  5: Given λ, solve (10) to compute $v_{i}$ for all *i*.  6: If $\sum_{i=1}^{N}v_{i}-\log\bar{P^{f}}>0$, then set $\lambda_{\min}=\lambda$, else set $\lambda_{\max}=\lambda$.  7:  **end while**.**Output:** Calculate $P_{i}^{f}$ (and also $\varepsilon_{i}$), and obtain the maximum $P^{d}$ in (6) and (7).

## 4. Numerical Results

In this section we present the numerical results with optimal cooperative sensing. For numerical results, the sensing duration τ is set at 1 msec and the channel bandwidth *B* is set at 6 MHz, both of which satisfy the test statistic of the sensing node in (1). The extensive numerical results are mainly based on the AND rule to verify the effect of *optimal thresholds* on both miss detection and false alarm performances so that no harmful interference as well as high spectral efficiency are guaranteed for PU and SUs, respectively. As aforementioned in Section 2.3, one may easily verify that our approach can be extended to the OR rule in a similar manner because of its symmetric mathematical structure as with the AND rule. Liu et al. [24] proposed a local optimal solution using iterative-threshold method, which is effective when SNRs are different. When average received SNRs are same for all cooperative sensing nodes, their sensing thresholds should be same [24]. However, when SNRs are same, the method in [24] cannot achieve the performance of the uniform threshold method because of its inherent limitation of iteration procedure. By contrast, our proposed algorithm can achieve a globally optimal solution whether SNRs are same or not. For the identical SNR case, we also verify that the uniform threshold method exactly matches our globally optimal solution.

**Figure 6:**
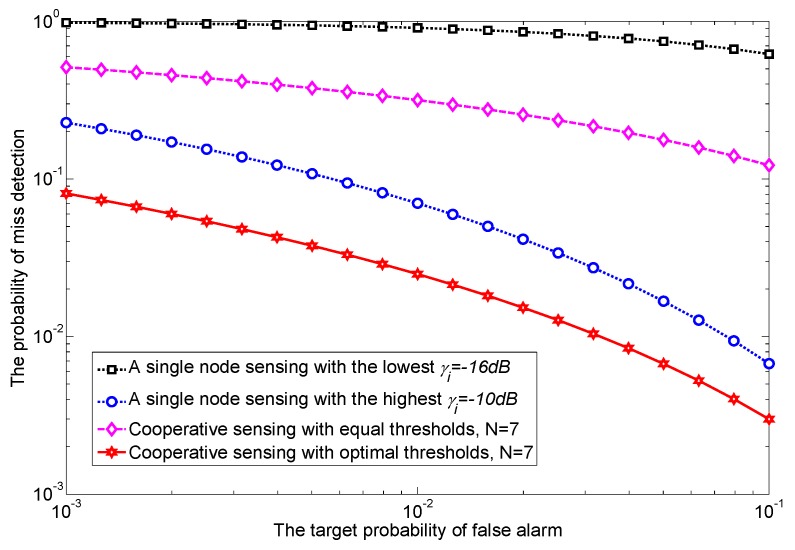
Receiver operating characteristic (ROC) curves for a single node sensing and cooperative sensing; different SNRs are considered in cooperation with both an equal threshold and optimal thresholds.

In Figure 6, ROC curves are presented for both a single node sensing and cooperative sensing with seven nodes, i.e., N=7. Each cooperative sensing node experiences its own average received SNR ${\bar{\gamma }}_{i}$, which is randomly selected in the interval [−10,−16] dB. We also set the ${\bar{\gamma }}_{i}$ of a single node sensing to be either −10 or −16 dB to represent the best and the worst case scenarios, respectively. As can be seen, although cooperative sensing with *equal threshold* (-⋄-) is obviously better than that of a single node sensing with ${\bar{\gamma }}_{i}=-16$ dB (-□-), it is interesting to observe that cooperative sensing (-⋄-) can be worse than single node sensing with ${\bar{\gamma }}_{i}=-10$ dB (-∘-). Namely, cooperation does *not* necessarily guarantee better sensing performance unless the detection thresholds are properly set. However, cooperative sensing with *optimal thresholds* (-✡-) significantly improves the sensing performance. Specifically, when compared to cooperative sensing with equal thresholds, under the target $\bar{P^{f}}$ is 0.01, and $P^{m}$ is reduced from 30% to 2% (and thus the corresponding $P^{d}$ is improved from 70% to 98%).

**Figure 7:**
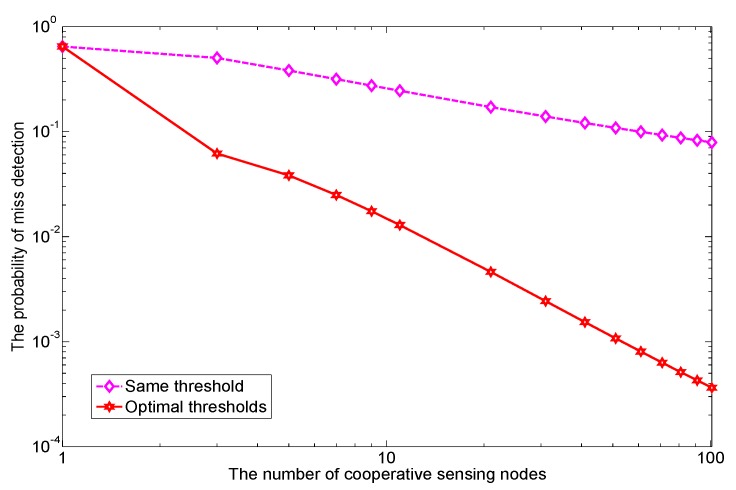
The probability of miss detection for two different thresholds methods are shown with the increasing number of cooperative sensing nodes; the target $\bar{P^{f}}=1\%$.

Next, to investigate the impact of *N* on optimal cooperative sensing performance, we compare the miss detection probabilities $P^{m}$ of the equal threshold method and the proposed optimal threshold in Figure 7. As can be seen, the more sensing nodes participate in cooperation, the lower the achieved $P^{m}$ is, in both cases. However, the slope of optimal threshold is much steeper than that of equal threshold. Furthermore, the miss detection probability is reduced by more than 100 times when *N* is 30 or more. Since the improvement is roughly proportional to *N*, we conclude that using optimized thresholds is essential for a large scale cooperative sensing, e.g., without optimal thresholds, many cooperative sensing nodes possibly lead to stagnant sensing performance.

**Figure 8:**
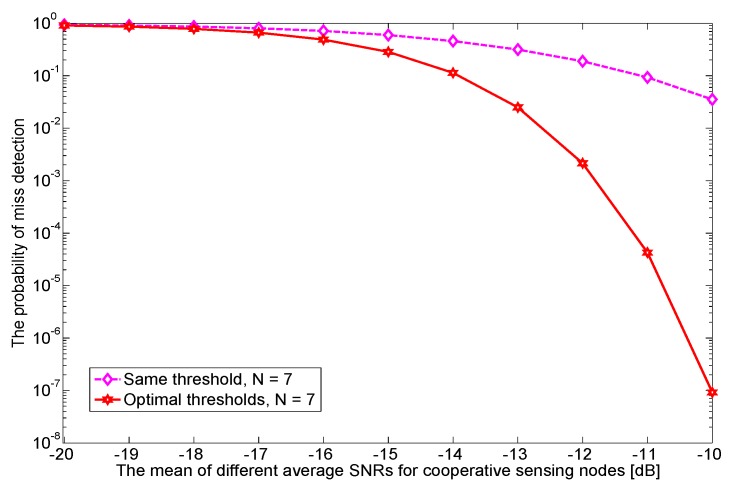
The probability of miss detection for two different thresholds methods are shown when the mean of different average SNRs of cooperative sensing nodes increases; the target $\bar{P^{f}}=1\%$ and N=7.

In Figure 8, we investigate the impact of the mean of different average SNRs $E[{\bar{\gamma }}_{i}]$ on $P^{m}$. When it is relatively low (below −17 dB), we do not observe a noticeable difference in $P^{m}$ between equal and optimal thresholds. However, as it grows, optimal thresholds can achieve much lower $P^{m}$ than that of equal thresholds. For example, a miss detection probability as low as $10^{-5}$ times can be achieved when $E[{\bar{\gamma }}_{i}]$ is −10 dB.

## 5. Conclusions

In this paper we proposed an optimal cooperative sensing solution using energy detection when sensing nodes experience different PU SNRs. Specifically, the fusion center and its cooperative sensing nodes used the hard decision under the Neyman–Pearson criterion. Despite the non-convexity of the problem, we provided a sufficient optimality condition that interestingly holds in the practical region of interest, and thus a globally optimal solution was obtained. Furthermore, utilizing optimal thresholds provides a gain of several orders of magnitude of cooperation in terms of miss detection probability compared with the conventional equal threshold under various scenarios of the target probability of false alarm, the number of cooperative sensing nodes, and the different average SNRs. Some practical extensions of the proposed scheme may include topics such as imperfect reporting channels, reduced reporting overheads, and energy-efficient sensing techniques in more general hard and soft decisions.

## Appendix A

**Proof** **of** **Proposition** **1.** For notational simplicity we define $a_{i}≜\frac{1}{\sqrt{1+\gamma_{i}}}$ and ${\tilde{\gamma }}_{i}≜\frac{\gamma_{i}\sqrt{\tau B}}{2}.$ Since (8) is not convex optimization, we again define $v_{i}≜\log Q\left(x_{i}\right)$ to make the inequality constraint of $v_{i}$ form a convex set. Using $x_{i}\;=\;{\left(\log Q\right)}^{-1}\left(v_{i}\right)$, the optimization in (8) becomes

(A1) $$\begin{array}{cc} \max_{v_{i},i\in \left\{1,\cdots ,N\right\}} & \sum_{i=1}^{N}\log Q\left(a_{i}\left({\left(\log Q\right)}^{-1}\left(v_{i}\right)-{\tilde{\gamma }}_{i}\right)\right),\\ s.t. & \sum_{i=1}^{N}v_{i}\leq \log\bar{P^{f}}. \end{array}$$

Here, we define a function f(t) as

$$f\left(t\right)≜Q\left(a_{i}\left({\left(\log Q\right)}^{-1}\left(t\right)-{\tilde{\gamma }}_{i}\right)\right).$$

Then, for the objective function of (A1), the pointwise sum $\sum_{i=1}^{N}\log f\left(v_{i}\right)$ is concave if $f(v_{i})$ is a log-concave function of $v_{i}$ for all *i* [29]. The log-concavity property of $f(v_{i})$ needs to hold for all *i*, so we now omit the subscript *i* for simplicity. To check whether f(v) is log-concave or not, we use the log-concave condition, e.g., the inequality is $f\left(v\right)f^{"}\left(v\right)\leq {f^{\prime }}^{2}\left(v\right)$ [29]. Although one may directly obtain the first and second derivatives of f(v) with respect to *v*, it is quite difficult to deal with ${\left(\log Q\right)}^{-1}\left(v\right)$ in f(v). Interestingly, we notice that it is possible to express both $f^{\prime }\left(v\right)$ and $f^{"}\left(v\right)$ as *a function of x*. Once we use a function defined as $z\left(t\right)≜Q(a\left(t-\tilde{\gamma }\right))$, then $f\left(v\right)=z\left(x\left(v\right)\right)=Q(a\left(x\left(v\right)-\tilde{\gamma }\right))$, where $x\left(v\right)={\left(\log Q\right)}^{-1}\left(v\right)$. To obtain $f^{\prime }\left(v\right)$ and $f^{"}\left(v\right)$, based on the chain rule $\frac{df(v)}{dv}=\frac{dz(x(v))}{dv}=\frac{dz(x)}{dx}\cdot \frac{dx}{dv}$ as well as the derivative formula ${\left(u^{-1}\right)}^{\prime }\left(v\right)=\frac{1}{u^{\prime }\left(u^{-1}\left(v\right)\right)}$ for any analytic function u:R→R [30], we have

$$\frac{dx}{dv}=\frac{Q(x)}{Q^{\prime }\left(x\right)},$$

which is *only a function of x.* Thus, we have $f^{\prime }\left(v\right)$ as follows:

(A2) $$f^{\prime }\left(v\right)=aQ^{\prime }\left(a\left(x-\tilde{\gamma }\right)\right)\frac{Q(x)}{Q^{\prime }\left(x\right)}.$$

In a similar way, we also have $f^{"}\left(v\right)$ as follows:

(A3) $$f^{"}\left(v\right)=a^{2}Q^{"}\left(a\left(x-\tilde{\gamma }\right)\right)\frac{Q{\left(x\right)}^{2}}{Q^{\prime }{\left(x\right)}^{2}}+aQ^{\prime }\left(a\left(x-\tilde{\gamma }\right)\right)\frac{Q(x)}{Q^{\prime }\left(x\right)}-aQ^{\prime }\left(a\left(x-\tilde{\gamma }\right)\right)Q^{"}\left(x\right)\frac{Q{\left(x\right)}^{2}}{Q^{\prime }{\left(x\right)}^{3}}.$$

Applying the log-concave condition $f^{"}\left(v\right)f\left(v\right)\leq {f^{\prime }}^{2}\left(v\right)$ with (A2) and (A3), the following long inequality condition is given by

(A4) $$\begin{array}{c} \left(a^{2}Q^{"}\left(a\left(x-\tilde{\gamma }\right)\right)\frac{Q{\left(x\right)}^{2}}{Q^{\prime }{\left(x\right)}^{2}}+aQ^{\prime }\left(a\left(x-\tilde{\gamma }\right)\right)\frac{Q(x)}{Q^{\prime }\left(x\right)}-aQ^{\prime }\left(a\left(x-\tilde{\gamma }\right)\right)Q^{"}\left(x\right)\frac{Q{\left(x\right)}^{2}}{Q^{\prime }{\left(x\right)}^{3}}\right)\\ \cdot Q\left(a\left(x-\tilde{\gamma }\right)\right)\leq a^{2}Q^{\prime }{\left(a\left(x-\tilde{\gamma }\right)\right)}^{2}\frac{Q{\left(x\right)}^{2}}{Q^{\prime }{\left(x\right)}^{2}}. \end{array}$$

After several steps of manipulations with $Q^{"}\left(t\right)=\frac{t}{\sqrt{2\pi }}\exp\left(\frac{-t^{2}}{2}\right)=-tQ^{\prime }\left(t\right)$ by (3), the inequality condition in (A4) boils down to:

$$x\left(1-a^{2}\right)+a^{2}\tilde{\gamma }+\frac{Q^{\prime }\left(x\right)}{Q(x)}\geq \frac{aQ^{\prime }\left(a\left(x-\tilde{\gamma }\right)\right)}{Q\left(a\left(x-\tilde{\gamma }\right)\right)}.$$

Finally, using a function $R\left(t\right)=\frac{Q^{\prime }\left(t\right)}{Q(t)}$, the inequality condition is given by

$$R\left(x\right)+x\geq a\left(R\left(a\left(x-\tilde{\gamma }\right)\right)+a\left(x-\tilde{\gamma }\right)\right).$$

With notations of $x_{i}$ and $y_{i}$, we obtain

$$R\left(x_{i}\right)+x_{i}\geq \frac{1}{\sqrt{1+\gamma_{i}}}\left(R\left(y_{i}\right)+y_{i}\right),\;\forall \;i\in \left\{1,\cdots ,N\right\},$$

which completes the proof. ☐
